# Discovery and Preclinical Validation of Salivary Transcriptomic and Proteomic Biomarkers for the Non-Invasive Detection of Breast Cancer

**DOI:** 10.1371/journal.pone.0015573

**Published:** 2010-12-31

**Authors:** Lei Zhang, Hua Xiao, Scott Karlan, Hui Zhou, Jenny Gross, David Elashoff, David Akin, Xinmin Yan, David Chia, Beth Karlan, David T. Wong

**Affiliations:** 1 School of Dentistry and Dental Research Institute, University of California Los Angeles, Los Angeles, California, United States of America; 2 Saul and Joyce Brandman Breast Center, Cedars-Sinai Medical Center, Los Angeles, California, United States of America; 3 Women's Cancer Research Institute, Cedars-Sinai Medical Center, Los Angeles, California, United States of America; 4 Department of Biostatistics, School of Public Health, University of California Los Angeles, Los Angeles, California, United States of America; 5 Department of Pathology and Laboratory Medicine, Jonsson Comprehensive Cancer Center, University of California Los Angeles, Los Angeles, California, United States of America; 6 Division of Hematology and Oncology, David Geffen School of Medicine, University of California Los Angeles, Los Angeles, California, United States of America; 7 Division of Head and Neck Surgery/Otolaryngology, David Geffen School of Medicine, University of California Los Angeles, Los Angeles, California, United States of America; 8 Henry Samueli School of Engineering and Applied Science, University of California Los Angeles, Los Angeles, California, United States of America; 9 Jonsson Comprehensive Cancer Center, University of California Los Angeles, Los Angeles, California, United States of America; Health Canada, Canada

## Abstract

**Background:**

A sensitive assay to identify biomarkers using non-invasively collected clinical specimens is ideal for breast cancer detection. While there are other studies showing disease biomarkers in saliva for breast cancer, our study tests the hypothesis that there are breast cancer discriminatory biomarkers in saliva using *de novo* discovery and validation approaches. This is the first study of this kind and no other study has engaged a *de novo* biomarker discovery approach in saliva for breast cancer detection. In this study, a case-control discovery and independent preclinical validations were conducted to evaluate the performance and translational utilities of salivary transcriptomic and proteomic biomarkers for breast cancer detection.

**Methodology/Principal Findings:**

Salivary transcriptomes and proteomes of 10 breast cancer patients and 10 matched controls were profiled using Affymetrix HG-U133-Plus-2.0 Array and two-dimensional difference gel electrophoresis (2D-DIGE), respectively. Preclinical validations were performed to evaluate the discovered biomarkers in an independent sample cohort of 30 breast cancer patients and 63 controls using RT-qPCR (transcriptomic biomarkers) and quantitative protein immunoblot (proteomic biomarkers). Transcriptomic and proteomic profiling revealed significant variations in salivary molecular biomarkers between breast cancer patients and matched controls. Eight mRNA biomarkers and one protein biomarker, which were not affected by the confounding factors, were pre-validated, yielding an accuracy of 92% (83% sensitive, 97% specific) on the preclinical validation sample set.

**Conclusions:**

Our findings support that transcriptomic and proteomic signatures in saliva can serve as biomarkers for the non-invasive detection of breast cancer. The salivary biomarkers possess discriminatory power for the detection of breast cancer, with high specificity and sensitivity, which paves the way for prediction model validation study followed by pivotal clinical validation.

## Introduction

Early detection of breast cancer is the key to positive, long-lasting outcomes, thus reducing the suffering and cost to society associated with the disease [1]. The high burden of breast cancer in women worldwide underscores the unmet potential of biomarker for early detection. A significant obstacle towards early detection of breast cancer is the development of methods that efficiently and accurately identify potentially affected individuals [2], [3].

Breast cancer has been among the earliest and most intensely-studied diseases using gene expression profiling and protein profiling technologies. The resulting molecular signatures help reveal the biological spectrum of breast cancers, providing diagnostic tools as well as prognostic and predictive gene signatures [4], [5]. Breast cancer detection is currently based on physical examination and imaging (mammography, ultrasound, and MRI) [6], although emerging methods include direct examination of the cytomorphology of exfoliated cells [7], and the molecular analysis of tumor biomarkers in nipple aspirate fluid or in ductal lavage [8], [9], [10]. In the last decade, biomarker discoveries for breast cancer detection have focused on blood and/or tissue, using proteomic [11], [12], [13], [14], [15], [16], transcriptomic [17], [18], [19], [20], [21], and genomic approaches [22], [23]. In comparison to prognostic biomarkers [24], [25], [26], the development of detection biomarkers has been limited, mainly due to a lack of sensitivity and specificity for this clinical context [2], [27], [28]. Most importantly, the use of tissue biomarkers for early detection will be limited to patients at very high risk because they rely on invasive procedures.

Recently, the study of salivary biomarkers has developed beyond oral diseases [29], [30], [31], [32] to systemic diseases [33], [34], broadening the potential for systemic disease detection [35], [36], [37], [38], [39]. Saliva-based translational research and technology is now at a mature juncture and can be evaluated to determine its utility for breast cancer detection. Explorative studies have evaluated the potential use of salivary proteins such as c-erbB-2, VEGF, EGF, and CEA in the initial detection and/or follow-up screening for the recurrence of breast cancer [33], [40], [41], [42], [43], [44]. However, these investigations were not based on biomarker discoveries from saliva specimens, rather they were testing blood biomarkers in saliva [45]. Here, we report the use of transcriptomic and proteomic approaches to discover and pre-validate biomarkers in saliva for the non-invasive detection of breast cancer. Our results demonstrate significant differences in salivary transcriptomic and proteomic profiles between breast cancer patients and controls. The discovered salivary biomarkers possess discriminatory power for the detection of breast cancer, with high specificity and sensitivity.

## Results

### Variation of salivary gene expression profiles and identification of mRNA biomarkers

**Figure 1:**
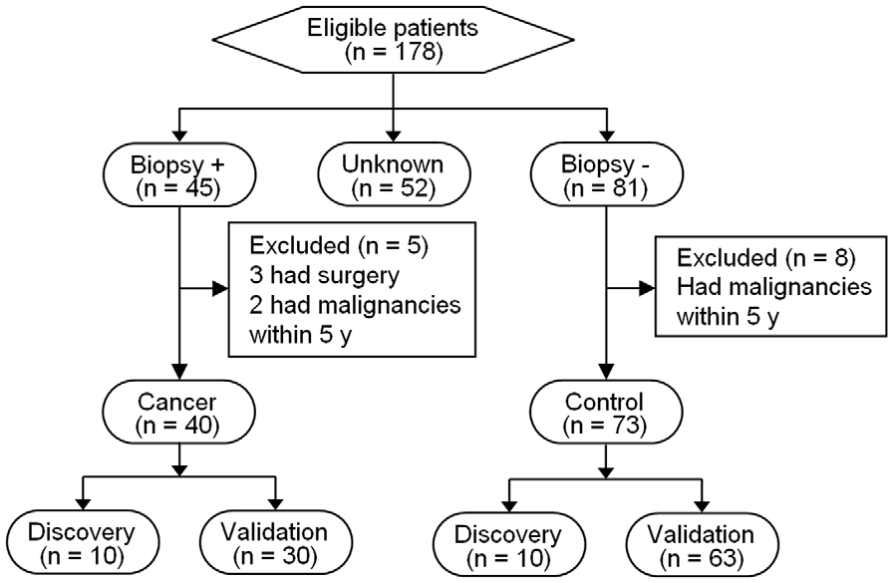
Schematic of the study design following the STARD reporting guideline.

Schematic of the study design and demographic information of all subjects used for the discovery and pre-validation phases are shown in Figure 1 and Table 1, respectively. Transcriptomic profiling identified 1402 genes exhibiting >2 fold up-regulation, and 2247 genes exhibiting >2 fold down-regulation, in the saliva of breast cancer patients, relative to the matched controls (n = 20, *P*<0.05). These transcriptomic changes were unlikely to be due to chance alone (*χ^2^* test, *P*<0.0001), considering the false positive rate with *P*<0.05. Using a predefined criterion of a change in regulation >2-fold, and a more stringent cutoff of *P*<0.01, 358 up-regulated and 943 down-regulated transcripts were identified in the saliva of breast cancer samples. RT-qPCR was performed to verify the microarray results on the discovery sample set (n = 20). The top 27 up-regulated candidates (Table S1) were selected based on p-value and fold-change (*P*<0.01, and >10-fold). The RT-qPCR results confirmed that the relative RNA expression levels of 11 up-regulated transcripts were consistent with the microarray. These verified transcriptomic biomarker candidates were then subjected to independent pre-validation by RT-qPCR using a cohort of 30 breast cancer patients and 63 controls (Figure 1). Eight up-regulated genes were pre-validated, showing significant differences between breast cancer and healthy controls (n = 93, Table 2).

**Table 1:** Demographic Information of All Subjects Used for the Discovery and Pre-validation Phases.

Demographic Variable	Characteristics	Discovery Phase			Pre-validation Phase		
		Breast Cancer (n = 10)	Healthy Control (n = 10)	p-value	Breast Cancer (n = 30)	Healthy Control (n = 63)	p-value
Age (y)	Mean ± SD	52.25±10.44	51.6±10.31	0.89	52.74±12.11	52.52±12.16	0.66
Gender	Female	10	10		30	63	
Ethnicity	Caucasian	5 (50%)	8 (80%)	0.44	19 (64.5%)	53 (83.9%)	0.07
	African-American	2 (20%)	0		1 (3.2%)	4 (6.5%)	
	Asian	0	0		6 (19.4%)	4 (6.5%)	
	Hispanic	3 (30%)	2 (20%)		0	1 (1.6%)	
	Other				4 (12.9%)	1 (1.6%)	
Smoking		3	3		10 (33.3%)	24 (38.1%)	1
HRT					10 (33.3%)		
Menopausal status	Pre	5	5		12 (40%)	32 (50.8%)	0.37
	Post	5	5		18 (60%)	31 (49.2%)	

**Table 2:** Validated biomarkers for breast cancer detection and effect of confounding factors (Pre-validation sample set n = 93).

Biomarker	P-value	cv.err	Age	Ethnicity	Menopausal Status	Smoking Status	HRT	Reported Relation to Breast Cancer or Other Cancers
*CSTA*	4.19E-13	0.333	0.16	0.78	0.24	0.95	0.08	[50]
*TPT1*	5.38E-05	0.251	0.30	0.60	0.13	0.87	0.17	[51]
*IGF2BP1*	2.57E-04	0.312	0.78	0.90	0.41	0.89	0.42	[52]
*GRM1*	6.57E-03	0.262	0.42	0.71	0.18	0.89	0.23	[53]
*GRIK1*	3.24E-02	0.237	0.70	0.80	0.36	0.88	0.20	[54]
*H6PD*	1.46E-03	0.262	0.57	0.73	0.30	0.76	0.21	[55]
*MDM4*	7.30E-04	0.297	0.55	0.79	0.27	0.89	0.25	[56]
*S100A8*	1.96E-03	0.272	0.54	0.86	0.31	0.88	0.22	[57]
CA6	1.70E-03	0.427	0.76	0.21	0.51	0.81	1.00	[58], [59]

NOTE: Eight mRNA biomarkers (in *italic*) were validated by RT-qPCR and one protein biomarker was validated by immunobloting using the validation sample set, including saliva from 30 breast cancer patients and 63 healthy control subjects. The Mann-Whitney rank sum test was used to determine marker validation. Possible confounding factors, including age, ethnicity, smoking status, menopausal status, and HRT treatment, were evaluated on the validated biomarkers by logistic regression model. Linear regression model was constructed for each marker and used the factors cancer/normal and one of the confounders. Abbreviations: cv.err: cross validation error rate.

### Variation of salivary proteomic profiles and identification of protein biomarkers

Proteomic profiling by 2D-DIGE revealed 35 up-regulated proteins/spots and 32 down-regulated proteins/spots in the saliva of breast cancer patients, relative to the matched controls (n = 20). Twenty spots, 14 up-regulated (>1.5 fold) and 6 down-regulated (>1.5 fold), were selected for protein identification, resulting in the identification of 10 up-regulated and 4 down-regulated proteins (Table S1). Four proteins (carbonic anhydrase VI (CA6), psoriasin, transthyretin, and cyclophilin A) with available antibodies were subjected to verification using immunoblot on the discovery sample set. The levels of CA6 and psoriasin between cancer and control samples showed significant differences (*p* = 0.012 and 0.014, respectively). These verified proteomic biomarker candidates were then independently validated by protein immunoblotting using the pre-validation cohort (30 breast cancer patients versus 63 controls). The level of CA6 showed a significant difference between breast cancer and healthy controls (n = 93, Table 2).

### Evaluation of the validated transcriptomic and proteomic biomarkers

**Figure 2:**
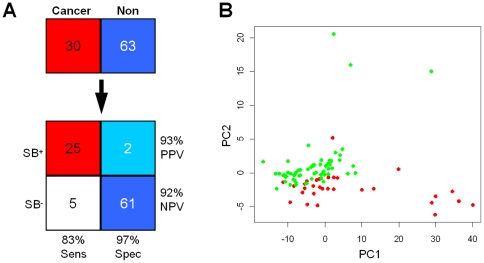
Clinical utility of the validated biomarkers. ***A***, Combination of nine validated biomarkers achieved a sensitivity of 83% (25 of 30 cancer subjects) with only a 3% false-positive rate (2 of the 63 control subjects). The shading of the contingency table boxes reflects the fraction of each samples type in each quadrant. ‘Cancer’ and ‘Non’ headings indicate subjects with and without cancer, respectively. SB+ and SB−, salivary biomarker test positive or negative, respectively; NPV, negative predictive value; PPV, positive predictive value; Sen, sensitivity; Spec, specificity. ***B***, Score plot of principle component analysis (PCA). Combining the nine validated biomarkers, the control subjects (green dots) separate from breast cancer patients (red dots), with cumulative proportions of 66.9% for PC1 and 21.6% for PC2.

Using logistic regression, the accuracy, sensitivity and specificity of 9-validated-biomarker combination on the pre-validation sample set (n = 93) were 92% (86 of 93), 83% (25 of 30) and 97% (61 of 63), respectively (Figure 2A). Principle component analysis (PCA) of this 9-biomarker combination could separate the breast cancer patients from the controls along the first principal component (*t*-test, *P*-value = 2.7E-15) (Figure 2B). None of the confounding factors (age, ethnicity, smoking status, menopausal status, and HRT treatment) significantly affected the validated biomarkers (Table 2). These indicate that cancer onset is a major source of variation in the expression of the validated biomarker. Furthermore, cross-disease comparisons showed that none of the validated mRNA biomarkers' expression was significantly altered in other salivary transcriptomic profiling studies, indicating their specificity for breast cancer detection (Table 3).

**Table 3:** Cross-disease comparisons of 8 validated salivary mRNA biomarkers.

Biomarker	Oral cancer	Lung cancer	Pancreatic cancer	Ovarian cancer	Diabetes	pSS	Breast cancer
*S100A8*	0.341	0.246	0.704	0.049	0.700	0.798	<0.001 *
*CSTA*	0.341	0.029	0.197	0.678	0.648	0.750	<0.001 *
*GRM1*	0.341	0.242	0.126	0.523	0.419	0.061	0.001 *
*TPT1*	0.341	0.112	0.558	0.090	0.454	0.855	<0.001 *
*GRIK1*	0.341	0.589	0.543	0.489	0.948	0.629	0.006 *
*H6PD*	0.343	0.517	0.475	0.293	0.330	0.101	<0.001 *
*IGF2BP1*	0.341	0.102	0.316	0.275	0.697	0.820	0.002 *
*MDM4*	0.341	0.011	0.154	0.455	0.088	0.168	0.001 *

NOTE: All analysis and comparison were based on microarray data. The validated mRNA biomarkers for breast cancer detection were checked against other microarray datasets (see text). Briefly, *t*-test p-values were calculated for all breast-cancer-validated genes in the other microarray datasets to check for significant variation (* after Bonferonni correction, *P*<0.006) between patients and controls in those diseases. Sample sizes of these microarray studies were 10 vs. 10 for oral cancer, 10 vs. 10 for lung cancer, 12 vs. 12 for pancreatic cancer, 11 vs. 11 for ovarian cancer, 13 vs. 13 for diabetes, 8 vs. 10 for pSS, and 10 vs. 10 for breast cancer.

## Discussion

Early detection of breast cancer offers the promise of easier treatment (smaller surgeries, less radiation or chemotherapy) and improved survival. Conventional screening (physical examination and mammography) has a less-than-desirable sensitivity and specificity [6]. There is a soaring need for new therapeutic strategies, as well as biomarkers that can achieve effective non-invasive early detection of breast cancer. Our long-term goal is to develop a saliva-based non-invasive tool for the early detection of breast cancer. We envision a clinical context in which a salivary test may enable clinicians to detect breast cancer earlier (by identifying patients warranting closer follow-up and additional imaging), and reduce the number of unnecessary biopsies (currently about 80% according to the American Cancer Society), in a cost-effective manner. The purpose of this study, which is an essential step toward attaining our long-range goal, is to evaluate the potential utility of salivary transcriptomes and proteomes for breast cancer detection. We applied two high-throughput technologies in order to assess 1) whether the salivary transcriptome and proteome profiles change with the onset of breast cancer, and 2) whether discriminatory biomarkers can be identified and validated. By addressing both questions, our profiling results, and further independent validation of the discovered biomarkers, will open new research directions and support the idea that saliva is a useful biomarker source for breast cancer detection.

The salivary transcriptome is a novel diagnostic alphabet we have explored for discovering breast cancer biomarkers. Salivary transcriptional profiling technology has been successfully applied for discovering detection biomarkers of resectable pancreatic cancer [34]. Consistent with that study, high-throughput profiling revealed significant variations in gene signature profiles between the breast cancer patients and the controls, demonstrating that the salivary transcriptome is an informative biomarker source for systemic cancer detection. The gene ontology analysis could categorize the 1301 up/down-regulated genes (>2 fold up/down-regulation, *P*<0.01) into various biological processes based on their known roles or functions. The 1301 genes were enriched in functions related to metabolic processes (35.46%), biological regulation (30.31%), and regulation of biological process (28.24%) (Figure S1). Based on the microarray data of 358 up-regulated transcripts (>2-fold change, *P*<0.01), breast cancer patients (n = 10) and matched controls (n = 10) could be classified into two distinct groups using unsupervised clustering, indicating the discriminatory power of salivary mRNA biomarkers (Figure S2). Our aim with transcriptomic profiling is not to identify large numbers of differentially expressed genes; rather we seek to find a small number of truly differentially expressed genes that can be validated. In this study, eight out of 27 top up-regulated transcripts (*P*<0.01, and >10-fold) were pre-validated using an independent cohort, yielding a validation rate of 29.6% that is similar to one of our previous study for pancreatic cancer (validation rate, 24.5%) [34].

Proteomic profiling, without independent validation, has been recently performed for discovering salivary biomarkers using stimulated whole saliva [46]. The results of our proteomic study overlap little with this previous proteomic profiling. This discrepancy could be due to the use of different disease types (invasive ductal carcinomas (IDC) versus ductal carcinoma in situ (DCIS)), different sample materials (unstimulated versus stimulated saliva), and different technical platforms. More importantly, we have conducted a pre–validation of the discovered protein biomarkers using an independent sample set. Interestingly, CA6, which was validated in our study, was also discovered in this previous proteomic profiling study using saliva samples from non-invasive breast cancer patients (DCIS) [46], indicating the potential of this biomarker for the early detection of breast cancer.

In order to obtain a more realistic estimate of the clinical utility of the validated biomarkers, and avoid the consequences of potential data overfitting, we employed leave-one-out cross-validation. The cross validation rate (cv.err) reflects a more accurate estimate of the true prediction accuracy of the biomarker. Except CA6, all comparisons have cross validation rates of ≤0.333, indicating that the validated biomarkers in general have high prediction accuracy (Table 2). Despite our moderate sample size, we appear to have identified biomarkers that significantly correlate with the presence of breast cancer.

Although the underlying relationships among systemic diseases and the saliva biomarkers are unclear, our recent study using mouse models has indicated that upon systemic disease development, cancer-specific changes occur in the salivary transcriptomic profiles [38]. Stimulation of the salivary glands by mediators released from remote tumors plays an important role in regulating the salivary surrogate biomarker profiles [38]. There may be extracellular communication between the ductal tissues of the breast and those of the salivary glands, since the histophysiology is very similar between these two distant tissues [46]. Interestingly, all validated biomarkers were previously implicated in breast cancer or other cancers (Table 2). Further investigation into the mechanism of salivary biomarkers for systemic cancers is warranted.

In summary, our study has identified transcriptomic and proteomic biomarkers in saliva that have the potential to impact current diagnostic triage for breast cancer. The salivary biomarkers' discriminatory power paves the way for a PRoBE-designed definitive validation study [45]. The critical feature of PRoBE design involves prospective clinical sample collection, before outcome ascertainment, from a study cohort that is relevant to the clinical application [45]. Any biomarker test intended for FDA approval and clinical use should incorporate the PRoBE principles as early as possible, as these principles eliminate potential biases commonly seen at the discovery stage.

## Materials and Methods

### Subject information and study design

This study, which was approved by the UCLA and Cedars-Sinai Medical Center Institutional Review Boards (#06-07-043 and #3870, respectively), began sample collection in February 2007. Written informed consents and questionnaire data sheets were obtained from all patients who agreed to serve as saliva donors. The saliva bank for breast cancer project at the UCLA Dental Research Institute, in collaboration with the Cedars-Sinai Medical Center, has collected 178 saliva samples from subjects recruited from the Saul and Joyce Brandman Breast Cancer Center. Of these, 113 samples, including 40 breast cancer patients and 73 healthy control individuals (Table 1), were used for the discovery and pre-validation phases of this study. Inclusion criteria of cancer patients consisted of a confirmed diagnosis of breast cancer. Exclusion criteria of cancer patients included therapy/surgery and/or a diagnosis of other malignancies within 5 years prior to saliva collection. Exclusion criteria of control patients included a diagnosis of any malignancies within 5 years prior to saliva collection (Figure 1). The information on patient characteristics, such as age, ethnicity, smoking history, menopausal status, and hormone replacement therapy (HRT), is presented in Table 1. Unstimulated saliva samples were consistently collected, stabilized, and preserved as previously described [34] (Figure S3). The sample supernatants were reserved at −80°C prior to assay.

This study consisted of a discovery phase, followed by an independent preclinical validation phase. Of the 113 samples, 10 breast cancer samples and 10 matched control samples were used for the discovery phase. All breast cancer cases were invasive ductal carcinoma (IDC), the most common type of breast cancer. Biomarkers identified from the discovery studies were first verified using the discovery sample set. An independent sample set, including 30 breast cancer patients and 63 controls, was used for the biomarker pre-validation phase (Figure 1).

### Salivary transcriptomic profiling and data analysis

RNA was isolated from 330 µl of saliva supernatant using MagMax™ Viral RNA Isolation Kit (Ambion, Austin, TX). This process was automated using KingFisher mL technology (Thermo Fisher Scientific, Waltham, MA), followed by TURBO™ DNase treatment (Ambion, Austin, TX). Extracted RNA was linearly amplified using the RiboAmp RNA Amplification kit (Molecular Devices, Sunnyvale, CA). After purification, cDNA was transcribed and biotinylated using GeneChip Expression 3′-Amplification Reagents for *in vitro* transcription labeling (Affymetrix, Santa Clara, CA). Chip hybridization and scanning were performed at the UCLA microarray core facility. Using the MIAME criteria [47], all Affymetrix Human Genome U133 Plus 2.0 Array data generated in this study were uploaded to the GEO database [48], accession number GSE20266.

The analysis was performed using R 2.7.0 with samr and ROC packages [49]. The Probe Logarithmic Intensity Error Estimation (PLIER) expression measures were computed after background correction and quantile normalization for each microarray dataset. Probeset-level quantile normalization was performed across all samples to make the effect sizes similar among all datasets. Finally, for every probeset, significance analysis of microarray (SAM) was applied to identify differential expression between the cancer and healthy control samples. The probesets were then ranked by the false discovery rate (FDR) corrected p-values.

### Preclinical validation of mRNA biomarkers using reverse transcription quantitative PCR (RT-qPCR)

The identified mRNA biomarkers were first verified by RT-qPCR using the discovery sample set (10 cancer versus 10 controls) as described previously [34]. RT-qPCR primers were designed using Primer Express 3.0 software (Applied Biosystems, Foster City, CA) (Table S2). All primers were synthesized by Sigma-Genosys (Woodlands, TX), and the amplicons were intron spanning whenever possible. RT-qPCR was carried out in duplicate. Verified biomarkers were then assayed by RT-qPCR in the set of 93 independent samples (30 breast cancer patients versus 63 controls). Raw data were normalized by subtracting GAPDH Ct values from the biomarker Ct values to generate ΔCt. The Mann-Whitney rank sum test was used for between-group biomarker comparisons.

### Salivary proteomic profiling and data analysis

Two-dimensional difference gel electrophoresis (2D-DIGE) was performed by Applied Biomics (Hayward, CA). Briefly, by taking equal amounts of protein from each sample, 10 cancer samples and 10 control samples were pooled separately, with each pool containing 250 µg of proteins. The proteins in each pool were precipitated by methanol and labeled with Cy3 and Cy5, respectively, and then combined for 2D-DIGE. After loading the labeled samples, the isoelectric focusing (IEF, pH 3–10) was run following the protocol provided by Amersham BioSciences (Piscataway, NJ). The immobilized pH gradient (IPG) strips were rinsed in the SDS-gel running buffer before transferring onto 13.5% SDS gels. The fold change of the protein expression levels was obtained from in-gel DeCyder analysis (Amersham BioSciences). Spots with a fold-change larger than 1.5 on the gel were subjected to in-gel trypsin digestion. The digested tryptic peptides were then mixed with CHCA matrix (alpha-cyano-4-hydroxycinnamic acid) and spotted into wells of a MALDI plate for MALDI-TOF MS identification (ABI4800, Applied Biosystems, Foster City, CA).

### Preclinical validation of protein biomarkers by immunoblotting

Protein immunoblotting was used to verify and validate the proteomic biomarker candidates. Reduced protein samples (15 µg total protein per lane) were loaded onto a 10% Bis-Tris gel and run at 150 Volt for one hour. Pre-stained protein standard (Invitrogen, USA) was used to track protein migration. The proteins were transferred to a nitrocellulose membrane and blocked for one hour in 5% non-fat dry milk. After further washes in TBST wash buffer, the membrane was incubated with the primary antibody (Lifespan bioscience, Seattle, WA) at room temperature for two hours. The membrane was then washed in TBST wash buffer before applying the secondary antibody (Anti-mouse IgG, peroxidase-linked species-specific whole antibody from sheep, GE healthcare, Piscataway, NJ) for one hour at room temperature. Finally, the membrane was washed in TBST wash buffer and visualized using the ECL Plus detection kit (GE Healthcare, Piscataway, NJ). The signal intensity of the bands was measured using Image J software (NIH, Bethesda, MD, USA). The intensity of a band representing the protein of interest was divided by the intensity of its corresponding β-actin expression on the same blot for normalization.

### Statistical analysis

Leave-one-out cross-validation was applied to assess the true accuracy of the model. In this procedure, each observation is iteratively taken out and the model is trained using all other observations. A prediction is then made on the left-out observation. The overall accuracy rate for each model is then the proportion of left out observations that are correctly predicted. To evaluate possible confounders for the markers versus cancer relationship, we examined factors such as age, ethnicity, smoking status, menopausal status, and HRT treatment. Linear regression model was constructed for each marker and used the factors cancer/normal and one of the above confounders.

The pre-validated breast cancer mRNA biomarkers were checked in other microarray studies that have been conducted in our laboratory on different diseases, including oral cancer [31], primary Sjögren's Syndrome (pSS) [30], pancreatic cancer [34], lung cancer, ovarian cancer, and type 2 diabetes. Briefly, P-value derived from Wilcoxon rank sum test were calculated for all breast-cancer-study-validated genes in other microarray datasets to check whether significant variation between breast cancer and controls also appeared in those disease datasets. After Bonferonni correction, variation was considered significant with p-values less than 0.006.

## Supporting Information

Figure S1 **Gene ontology analysis of the up/down-regulated genes (1301 genes, >2 fold up/down-regulation, P<0.01)**.
(PDF)

Figure S2 **Heatmap of 358 up-regulated transcripts based on microarray data (>2-fold change, *P*<0.01)**. Hierarchical clustering and gene function enrichment was performed using Euclidean distance metric and Average linkage method (unsupervised clustering). Breast cancer patients (n = 10) and healthy controls (n = 10) could be classified into distinct groups, indicating the discriminatory power of salivary mRNA biomarkers. The GEO database access number of all microarray experiments is GSE20266.
(PDF)

Figure S3 **Protocol for saliva collection**.
(PDF)

Table S1 **Biomarker candidates selected from transcriptomic and proteomic profiling**.
(PDF)

Table S2 **Primers of 11 verified transcripts and *GAPDH***.
(PDF)
