# Supplementary figures and images for: Discovery and Preclinical Validation of Salivary Transcriptomic and Proteomic Biomarkers for the Non-Invasive Detection of Breast Cancer
Source: PLoS One. 2010 Dec 31;5(12):e15573. doi: 10.1371/journal.pone.0015573 (PMC3013113; doi:10.1371/journal.pone.0015573)

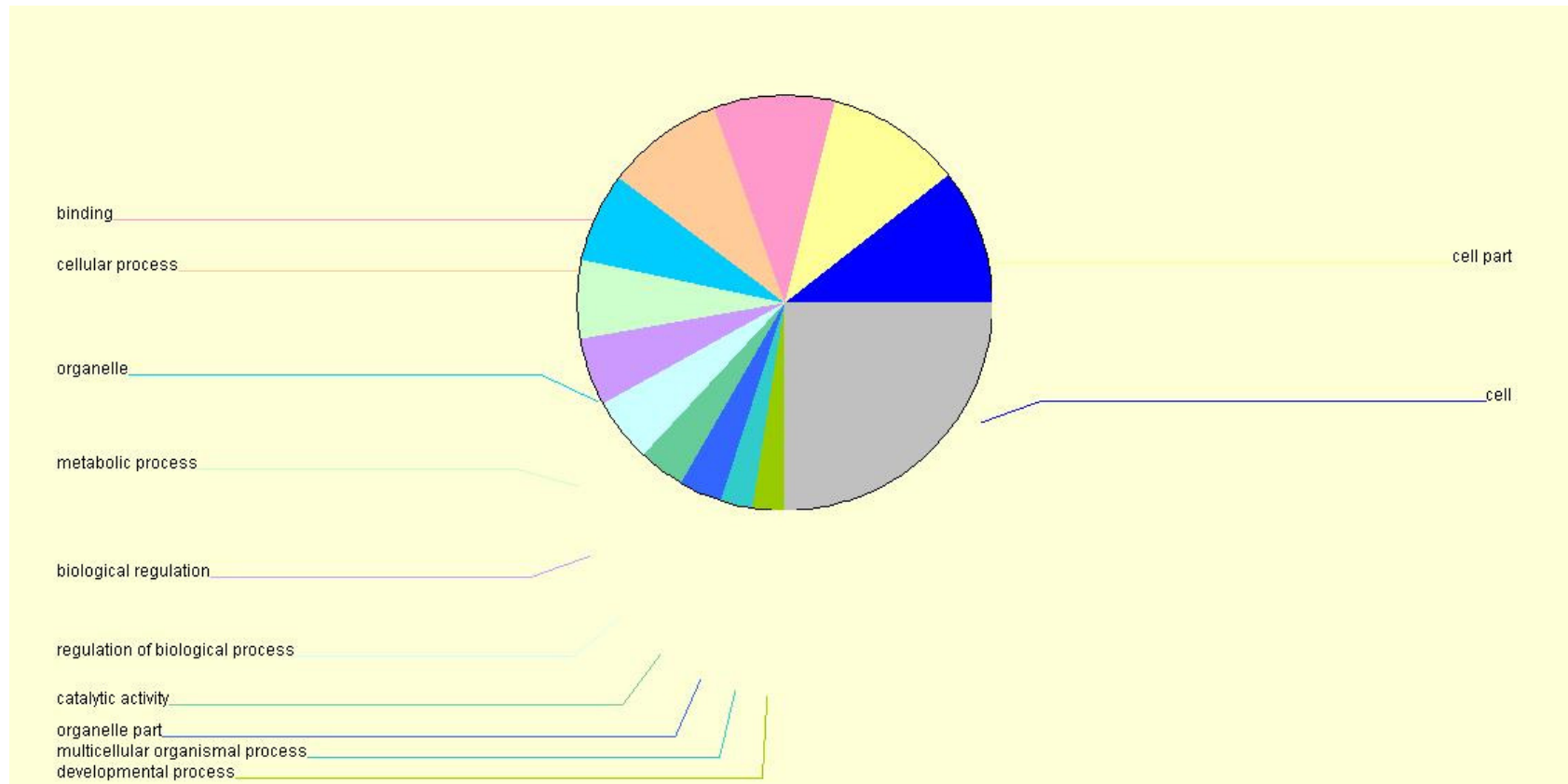

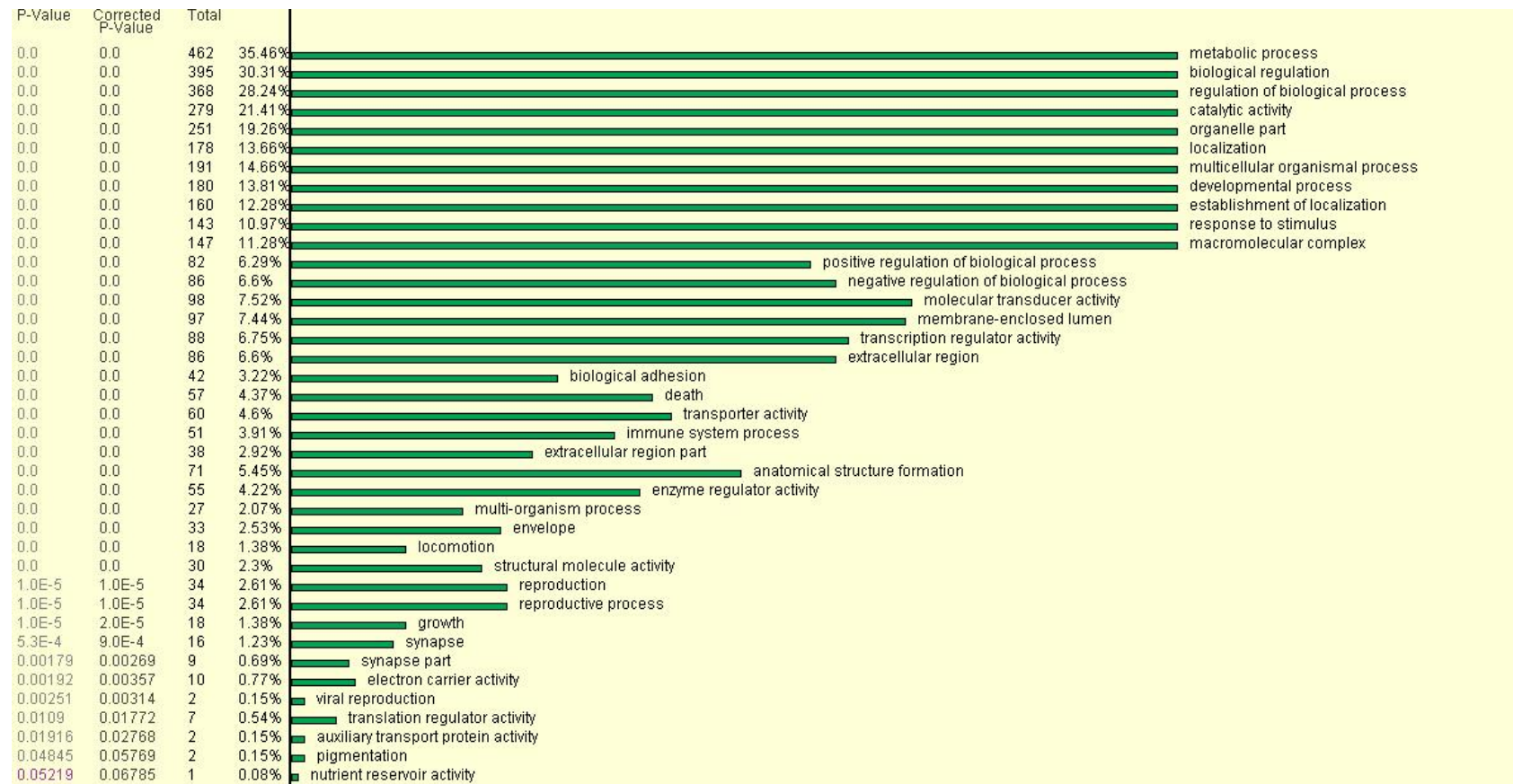

Supplement: Figure S1 — Gene ontology analysis of the up/down-regulated genes (1301 genes, >2 fold up/down-regulation, P<0.01). (PDF) [file pone.0015573.s001.pdf]

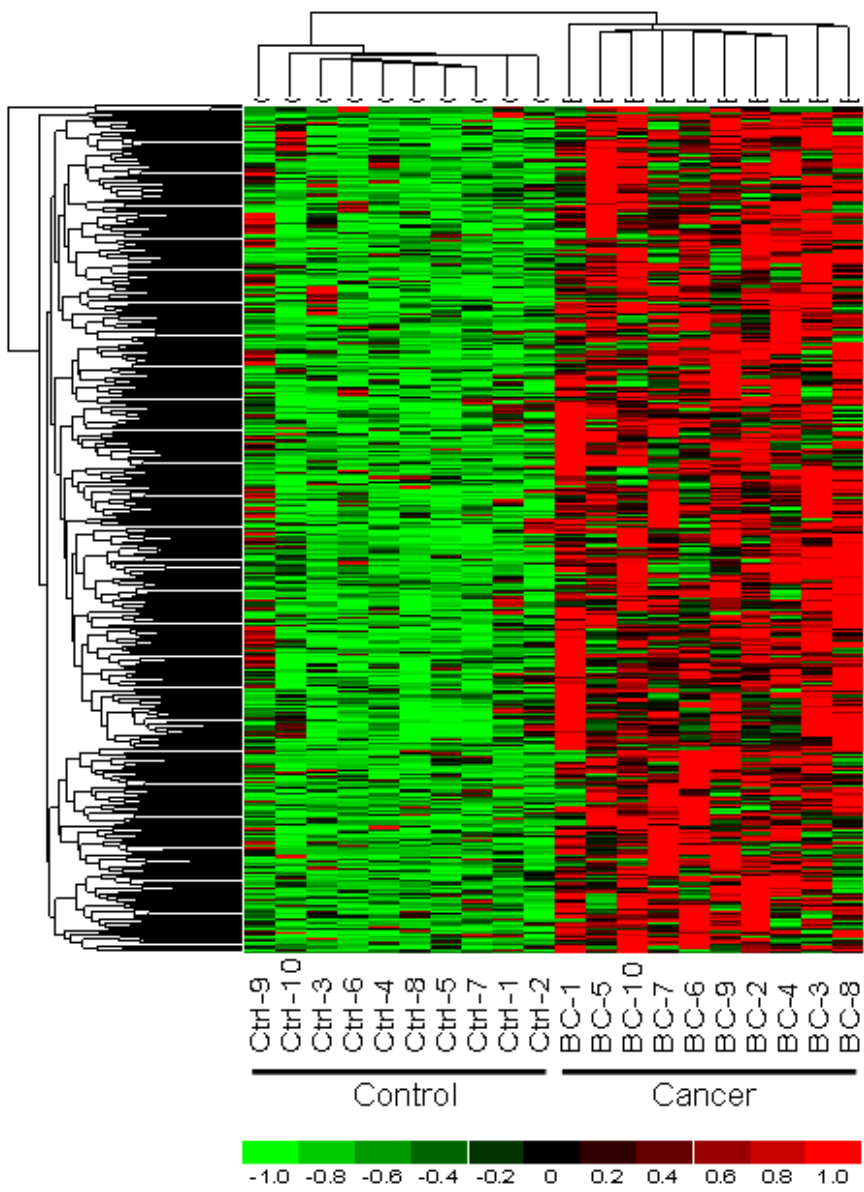

Supplement: Figure S2 — Heatmap of 358 up-regulated transcripts based on microarray data (>2-fold change, P<0.01). Hierarchical clustering and gene function enrichment was performed using Euclidean distance metric and Average linkage method (unsupervised clustering). Breast cancer patients (n = 10) and healthy controls (n = 10) could be classified into distinct groups, indicating the discriminatory power of salivary mRNA biomarkers. The GEO database access number of all microarray experiments is GSE20266. (PDF) [file pone.0015573.s002.pdf]
