# Supplementary material for: Discovery and Preclinical Validation of Salivary Transcriptomic and Proteomic Biomarkers for the Non-Invasive Detection of Breast Cancer
Source: PLoS One. 2010 Dec 31;5(12):e15573. doi: 10.1371/journal.pone.0015573 (PMC3013113; doi:10.1371/journal.pone.0015573)
Supplement: Figure S3 — Protocol for saliva collection. (PDF) [file pone.0015573.s003.pdf]

Protocol for Saliva Collection

1. Make appointment with subject, preferably 9-11 am, and ask subject to refrain from eating, drinking or using oral hygiene products for at least 1 hour prior to collection.
2. Ask subject to rinse mouth with water.
3. 5 minutes after oral rinse, ask subject to spit into a 50 ul Falcon tube kept **on ice**, for example in a styrofoam coffee cup filled with ice. Remind subject not to cough up mucus. Collect 5 ml of saliva for not more than 30 minutes.
4. Centrifuge at 2,600g for 15 min at 4°C. If incomplete separation has occurred, spin another 20 minutes.
5. Collect as much supernatant as possible into a 15ul Falcon tube and mix briefly. Divide supernatant into 2 parts: pipette 1 ml into a 2 ml Cryo tube and treat as Part 1 for protein studies. Treat the rest of the supernatant as Part 2 for RNA studies and aliquot 1ml fractions into 2ml Cryo tubes. Add appropriate reagents to Part 1 and 2 as indicated below. Samples should be processed and frozen within 30 minutes from the time of collection. Save the pellet into a Cryo tube and store at -80°C.
6. In this manuscript, we used the Part 1 collection for the proteomic study and Part 2 collection for the transcriptomic study.

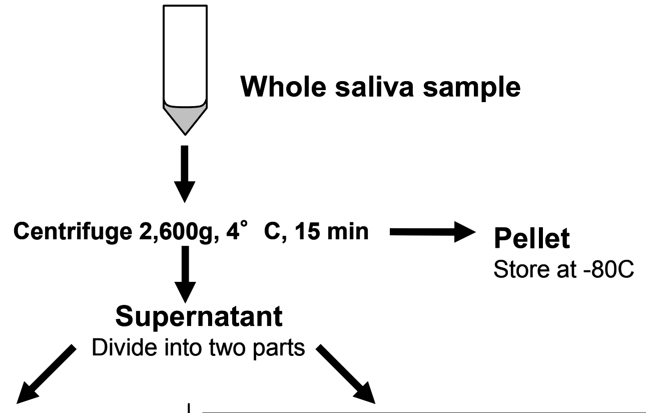

Part 1 (PROTEIN) :

1. Per ml of saliva supernatant add :
   - 1 ul Aprotinin stock 10 mg/ml
   - 3 ul Na_3OV_4 stock 400 mM
   - 10 ul PMSF stock 10 mg/ml

   Invert gently
2. Mark sample as: SalSup Pro
   CS-BC-001, 002, etc.
   Date of collection
3. Insert a yellow tube top. Freeze at -80°C right away

Part 2 (RNA) :

1. Per ml of saliva supernatant add : 5 ul (100 Units) of Superase Inhibitor “SI”.
2. Mark sample as: SalSup RNA
   CS-BC-001, 002, etc.
   Date of collection
3. Insert a white tube top. Freeze at -80°C right away
