# Supplementary material for: Discovery and Preclinical Validation of Salivary Transcriptomic and Proteomic Biomarkers for the Non-Invasive Detection of Breast Cancer
Source: PLoS One. 2010 Dec 31;5(12):e15573. doi: 10.1371/journal.pone.0015573 (PMC3013113; doi:10.1371/journal.pone.0015573)
Supplement: Table S1 — Biomarker candidates selected from transcriptomic and proteomic profiling. (PDF) [file pone.0015573.s004.pdf]

**Table S1.** Biomarker Candidates Selected from Transcriptomic and Proteomic Profiling				
**Gene/Protein Symbol**	**Gene/Protein Name**	**Platform**	**Representative Public ID**	**Go biological Process Term**
RGS13	regulator of G-protein signaling 13	Transcriptomic	BC036950	G-protein coupled receptor protein signaling pathway
S100A8	S100 calcium binding protein A8	Transcriptomic	NM_002964	inflammatory response
NSUN5	NOL1/NOP2/Sun domain family, member 5	Transcriptomic	NM_018044	
CSTA	cystatin A (stefin A)	Transcriptomic	NM_005213	negative regulation of peptidase activity, peptide cross-linking, keratinocyte differentiation
ATXN3	ataxin 3	Transcriptomic	AI888099	nucleotide-excision repair, transcription, synaptic transmission, nervous system development, cell death, regulation of transcription
LOR	loricrin	Transcriptomic	NM_000427	peptide cross-linking, keratinocyte differentiation, keratinization
XPO7	exportin 7	Transcriptomic	NM_015024	intracellular protein transport, protein transport, mRNA transport, transmembrane transport, intracellular protein transmembrane transport
MBD1	methyl-CpG binding domain protein 1	Transcriptomic	NM_015845	transcription, transcription from RNA polymerase II promoter, negative regulation of transcription, regulation of transcription
PRCC	papillary renal cell carcinoma (translocation-associated)	Transcriptomic	BC004913	
ITGAX	integrin, alpha X (complement component 3 receptor 4 subunit)	Transcriptomic	M81695	cell adhesion, cell adhesion, integrin-mediated signaling pathway, organ morphogenesis
IGH	immunoglobulin heavy locus	Transcriptomic	L23515	immune response, protein-chromophore linkage
TPT1	tumor protein, translationally-controlled 1	Transcriptomic	BG498776	calcium ion transport, cellular calcium ion homeostasis, anti-apoptosis, regulation of apoptosis
GM2A	GM2 ganglioside activator	Transcriptomic	AL513583	lipid metabolic process, sphingolipid metabolic process, ganglioside catabolic process, lipid transport and storage
MYO1F	myosin IF	Transcriptomic	BF740152	negative regulation of cell adhesion, positive regulation of cell migration, regulation of

				actin cytoskeleton organization, neutrophil degranulation, regulation of innate immune response, defense response to Gram-positive bacterium
PAK7	p21 protein (Cdc42/Rac)-activated kinase 7	Transcriptomic	BF056517	protein amino acid phosphorylation, anti-apoptosis
SPTB	spectrin, beta, erythrocytic	Transcriptomic	BG223341	actin filament capping
GRIK1	glutamate receptor, ionotropic, kainate 1	Transcriptomic	U16125	ion transport, glutamate signaling pathway, synaptic transmission, nervous system development
GRHPR	glyoxylate reductase/hydroxypyruvate reductase	Transcriptomic	AK024386	transcription, excretion, metabolic process, oxidation reduction
CLDN15	claudin 15	Transcriptomic	NM_014343	calcium-independent cell-cell adhesion
ALLC	allantoicase	Transcriptomic	NM_018436	
H6PD	hexose-6-phosphate dehydrogenase (glucose 1-dehydrogenase)	Transcriptomic	AK024548	carbohydrate metabolic process, glucose metabolic process, pentose-phosphate shunt, metabolic process, oxidation reduction
IGF2BP1	insulin-like growth factor 2 mRNA binding protein 1	Transcriptomic	AF198254	RNA localization, regulation of translation, regulation of mRNA stability involved in response to stress, negative regulation of translation, regulation of cytokine biosynthetic process
PDCD1LG2	programmed cell death 1 ligand 2	Transcriptomic	AF329193	immune response, regulation of T cell proliferation
HAS3	hyaluronan synthase 3	Transcriptomic	AI739514	carbohydrate metabolic process
MDM4	Mdm4 p53 binding protein homolog (mouse)	Transcriptomic	AA745971	negative regulation of transcription from RNA polymerase II promoter, protein complex assembly, apoptosis, cell proliferation
IGF1R	insulin-like growth factor 1 receptor	Transcriptomic	BF347362	protein amino acid phosphorylation, anti-apoptosis, immune response, positive regulation of cell proliferation, insulin receptor signaling pathway, insulin-like growth factor receptor signaling pathway
RGS8	regulator of G-protein signaling 8	Transcriptomic	R37101	negative regulation of signal transduction
CA6	Carbonic Anhydrase VI	Proteomic	EAW71606	one-carbon metabolic process
GATC	Glutamyl-tRNA(Gln) amidotransferase	Proteomic	YP_001209858	mRNA splice site selection, mRNA processing, mRNA processing, regulation of translational fidelity, RNA splicing
LCN1	Lipocalin 1 precursor	Proteomic	NP_002288	proteolysis, transport, response to stimulus, sensory perception of taste

TTR	Transthyretin	Proteomic	3CFM_A	thyroid hormone generation, transport, transport
PRDX5	Putative peroxisomal antioxidant enzyme	Proteomic	AAF17200	inflammatory response, response to oxidative stress, cellular response to reactive oxygen species, negative regulation of apoptosis, cell redox homeostasis, oxidation reduction
PPIA	Cyclophilin A	Proteomic	5CYH_A	protein folding, provirus integration, initiation of viral infection, interspecies interaction between organisms, regulation of viral genome replication
S100A7	Psoriasin	Proteomic	AAA60210	response to reactive oxygen species, angiogenesis, epidermis development, keratinocyte differentiation, innate immune response, defense response to Gram-negative bacterium, sequestering of metal ion
HBA2	Alpha 2 globin	Proteomic	NP_000508	transport, oxygen transport
KRT9	Keratin 9	Proteomic	EAW60744	spermatogenesis, epidermis development, skin development, intermediate filament organization
KRT16	Type I keratin 16	Proteomic	AAB35421	cytoskeleton organization, cell proliferation, epidermis development, intermediate filament cytoskeleton organization
KRT14	Keratin 14	Proteomic	NP_000517	epidermis development, response to zinc ion, response to ionizing radiation, epithelial cell differentiation, intermediate filament bundle assembly
SCFD1	Vesicle transport-related protein	Proteomic	BAD96540	transport, retrograde vesicle-mediated transport, response to stress, response to toxin, regulation of ER to Golgi vesicle-mediated transport
ALKBH8	Alkylation repair homolog 8	Proteomic	XP_944595	metabolic process
TXNDC5	thioredoxin domain containing 5 (endoplasmic reticulum)	Proteomic	EAW55223	anti-apoptosis, cell redox homeostasis
