# Supplementary material for: Discovery and Preclinical Validation of Salivary Transcriptomic and Proteomic Biomarkers for the Non-Invasive Detection of Breast Cancer
Source: PLoS One. 2010 Dec 31;5(12):e15573. doi: 10.1371/journal.pone.0015573 (PMC3013113; doi:10.1371/journal.pone.0015573)
Supplement: Table S2 — Primers of 11 verified transcripts and GAPDH. (PDF) [file pone.0015573.s005.pdf]

**Table S2**. Primers of 11 verified transcripts and *GAPDH*		
**Gene symbol**	**Primer name**	**Primer sequences (5'-3')**
ATXN3	ATXN3-OF	GAAAAACAGCAGCAAAAGCA
	ATXN3-IF	GGGGGACCTATCAGGACAGA
	ATXN3-IR	CAAGTGCTCCTGAACTGGTG
	ATXN3-OR	CCAAGTGCTCCTGAACTGGT
GRIK1	GRIK1-OF	CCGGACTGGTCCTTTCTGTA
	GRIK1-IF	CCGGACTGGTCCTTTCTGTA
	GRIK1-IR	AGCGTTGAAAGAGAGACACTG
	GRIK1-OR	CAGTGAGATTCCCAGTTCTTCC
GRM1	GRM1-OF	GCAGGGAATGCCAATTCTAA
	GRM1-IF	TGGCAAGTCTGTGTCATGGT
	GRM1-IR	GCCACATATGCTGTCCCTTG
	GRM1-OR	GCCGTCTCATTGGTCTTCAC
TPT1	TPT1-OF	TACCGTGAGGATGGTGTGAC
	TPT1-IF	CAAATGTGGCAATTATTTTGGA
	TPT1-IR	GATGACAAGCAGAAGCCAGTT
	TPT1-OR	GATGACAAGCAGAAGCCAGT
RGS13	RGS13-OF	CTCACGGTGGAGCAGAATTT
	RGS13-IF	CTCACGGTGGAGCAGAATTT
	RGS13-IR	GGGACTGTGGCTGGATGTAA
	RGS13-OR	TGGGTTCCTGAATGTTCCTG
S100A8	S100A8-OF	TCAGGAAAAAGGGTGCAGAC
	S100A8-IF	TCAGGAAAAAGGGTGCAGAC
	S100A8-IR	TGGAAGTTAACTGCACCATCA
	S100A8-OR	ACGCCCATCTTTATCACCAG
CLDN15	CLDN15-OF	TTGTACCCCGGAACCAAGTA
	CLDN15-IF	CGGAACCAAGTACGAGCTG
	CLDN15-IR	CACCCAGGATGGAGATCAGT
	CLDN15-OR	CTGGGTCCTCGTCAGAGC
IGF2BP1	IGF2BP1-OF	AGAATTTGACGGCAGCTGAG
	IGF2BP1-IF	CCAGGTCATCGTGAAAATCA
	IGF2BP1-IR	ATCTTCCGTTGAGCCATCTG
	IGF2BP1-OR	ATGTCTCGGATCTTCCGTTG
CSTA	CSTA-OF	ACGGAAAATTGGAAGCTGTG
	CSTA-IF	CATTAAGGTACGAGCAGGTGA
	CSTA-IR	TTTGTCCGGGAAGACTTTTG
	CSTA-OR	TTTGTCCGGGAAGACTTTTG
MDM4	MDM4-OF	GTGGCAGTGTACTGAATGCAA
	MDM4-IF	TGGCAGTGTACTGAATGCAA
	MDM4-IR	AAGGCCCAACAACGAAAAC
	MDM4-OR	TCAGACGTGGAGAGAGAATGG
H6PD	H6PD-OF	GGCACAAGCTTCAGGTCTTC

	H6PD-IF	GTCGTGGGCCAGTACCAGT
	H6PD-IR	GTGGAAGCTGTCTGGCTTCT
	H6PD-OR	GTGGAAGCTGTCTGGCTTCT
GAPDH	GAPDH-OF	CATTGCCCTCAACGACCACTT
	GAPDH-IF	ACCACTTTGTCAAGCTCATTTCCT
	GAPDH-IR	CACCCTGTTGCTGTAGCCAAAT
	GAPDH-OR	ATGTGGGCCATGAGGTCCA

NOTE: OF = Outer forward, IF = Inner forward, IR = Inner reverse, OR = Outer reverse. All primers were designed using Primer Express 3.0 software (Applied Biosystems, FosterCity, CA). The specificity of primers was checked using NCBI's GenBank BLAST search.
